# Do We Need a Gwent Tier 3 FCAMHS Neurodevelopmental Assessment Pathway?

**DOI:** 10.1192/bjo.2025.10529

**Published:** 2025-06-20

**Authors:** Andrew Todd, Michael Jones, Adam Edwards, Nicola Crawley, Danielle John

**Affiliations:** 1 Aneurin Bevan University Health Board, Caerleon, United Kingdom; 2 Monmouthshire & Torfaen Youth Offending Service, Pontypool, United Kingdom; 3 Newport Youth Offending Service, Newport, United Kingdom; 4 Blaenau Gwent & Caerphilly Youth Offending Service, Blackwood, United Kingdom

## Abstract

**Aims:** There are high rates of Neurodevelopmental Disorders (NDD) and mental health needs in young people who engage in offending behaviours. Incompletely assessed development can be a factor in engaging with legal proceedings and with those involved in their care. Young people open to the three Gwent Youth Offending Services (YOS) and Tier 3 Gwent Forensic Child & Adolescent Mental Health Service (FCAMHS) have previously been identified as having traits indicative of an underlying NDD. No specific ND assessment pathways exist within YOS for suspected Autism Spectrum Disorder (ASD) and/or Attention Deficit Hyperactivity Disorder (ADHD); those requiring assessment are referred to the CAMHS ND Team. Some cases are Not in Education, Employment or further Training (NEET), may not reside with family, and can struggle to engage in typical referral pathways.

An evaluation is taking place regarding the use of existing Gwent CAMHS and YOS resources for piloting a Tier 3 Gwent Forensic CAMHS Neurodevelopmental Assessment Pathway (FCAMHS NDAP).

**Methods:** Gwent FCAMHS is a small Multidisciplinary Team (MDT):

One part-time Consultant Forensic Child & Adolescent Psychiatrist.

Four Psychiatric Nurses.

One part-time Forensic Psychologist.

Between December 2023–December 2024 the number of YOS cases referred for and receiving ND assessment were logged. Assessment outcomes were considered with ongoing discussions with each of the YOS to ascertain ongoing roll-out of a pilot FCAMHS NDAP.

**Results:** Between December 2023–December 2024:

7 cases were referred (5 for possible ADHD, 2 for ASD).

6 of the referrals were not in mainstream education or NEET.

6 ND assessments were completed.

5 resulted in an NDD diagnosis (ADHD=4, ASD=1).

2 cases necessitated ADOS-2 assessment and 1 case was assessed using the Qb Test protocol.

Upon diagnosis, 2 elected to trial ADHD medication.

Referrals originated from all three YOS in Gwent: Newport (2), Blaenau Gwent-Caerphilly (4) and Monmouthshire-Torfaen (1).

**Conclusion:** Cases were diverted from the CAMHSND waiting list to FCAMHS. YOS support in case engagement and obtaining information was vital for the assessments. YOS managers recognise the role an FCAMHS NDAP has for some cases in the Gwent YOS who would struggle to engage in the typical pathway. Requests have been received for YOS staff training in NDD.

A formal roll-out of a Gwent FCAMHS NDAP pilot with specific inclusion criteria has been agreed with the YOS teams and Gwent FCAMHS, commencing January 2025. Quality Improvement methodology is utilised and training packages for YOS staff are being developed.

